# Intestinal schistosomiasis and geohelminths of Ukara Island, North-Western Tanzania: prevalence, intensity of infection and associated risk factors among school children

**DOI:** 10.1186/s13071-014-0612-5

**Published:** 2014-12-23

**Authors:** Moshi Mugono, Evelyne Konje, Susan Kuhn, Filbert J Mpogoro, Domenica Morona, Humphrey D Mazigo

**Affiliations:** School of Public Health, Catholic University of Health and Allied Sciences, P.O. Box 1464, Mwanza, Tanzania; Department of Paediatrics, Section of Infectious Diseases, University of Calgary, Calgary, Canada; Department of Medical Parasitology and Entomology, School of Medicine, Catholic University of Health and Allied Sciences, P.O. Box 1464, Mwanza, Tanzania

**Keywords:** *Schistosoma mansoni*, Soil-transmitted helminths, Ukara Island, North-Western Tanzania

## Abstract

**Background:**

*Schistosoma mansoni* and soil-transmitted helminths (STH) are among the most prevalent and highly neglected tropical diseases in Tanzania. However, little is known on the distribution of these infections in rural settings, especially in the island areas on Lake Victoria. Identifying the local risk factors of *S. mansoni* and soil-transmitted helminths is one step towards understanding their transmission patterns and will facilitate the design of cost-effective intervention measures. The present study was therefore conducted to determine the prevalence, intensity of infection and risk factors associated with *S. mansoni* and soil-transmitted helminth infections among school children in Ukara Island.

**Methods:**

This was a cross sectional study which enrolled 774 school children aged 4-15 years in 5 primary schools in Ukara Island, North-Western Tanzania. Single stool samples were collected, processed using the Kato Katz technique and examined for eggs of *S. mansoni* and geohelminths under a light microscope. A pre-tested questionnaire was used to collect socio-demographic information.

**Results:**

Overall, 494/773 (63.91%, 95% CI; 45.19-90.36) of the study participants were infected with *S. mansoni* and the overall geometrical mean eggs per gram (GM-epg) of feaces were 323.41epg (95% CI: 281.09 – 372.11). The overall prevalence of soil-transmitted helminth (STH) was 6.73% (n = 52/773, 95% CI = 4.39 – 10.32) with the most prevalent species being hookworms, 5.69% (n = 44/773, 95% CI; 3.68 – 8.79). Location of school in the study villages (*P <* 0.0001), parent occupation, fishing (*P <* 0.03) and reported involvement in fishing activities (*P <* 0.048) remained significantly associated with the prevalence and intensity of *S.mansoni* infection.

**Conclusion:**

*Schistosoma mansoni* infection is highly prevalent in the islands whereas the prevalence of soil-transmitted helminths is low. The risk of infection with *S. mansoni* and the intensity of infection increased along the shorelines of Lake Victoria. These findings call for the need to urgently implement integrated control interventions, starting with targeted mass drug administration.

**Electronic supplementary material:**

The online version of this article (doi:10.1186/s13071-014-0612-5) contains supplementary material, which is available to authorized users.

## Background

The Sub-Saharan Africa (SSA) region is endemic to schistosomiasis and soil-transmitted helminth (STH), with many areas reaching high transmission levels [1,2]. Of the 249 millions cases of schistosomiasis occurring in 78 endemic countries of the world, 90% (192 million cases) occurs in SSA [1,2]. An estimated 779 million individuals live in areas potentially risky for the transmission of schistosomiasis [2]. In the SSA region, *S. mansoni* and *S. haematobium* are known to cause intestinal and urogenital schistosomiasis, with the former being focally distributed and the later widely distributed [1-3]. For the soil-transmitted helminths (STH), an estimated 198 million people are infected with hookworm, 173 million with *A. lumbricoides* and 162 million with *T. trichura* in SSA [1,4]. Chronic infection with soil-transmitted helminths results into malnutrition, micronutrient deficiencies, poor cognitive function and school absenteeism [5], whereas chronic infection with *S. mansoni* results in hepatomegaly, hepatosplenomegaly and poor growth in children [5]. Despite the serious health impact resulting from these infections and their predominance in areas of poverty, their geographical distribution especially in rural remote areas of SSA, remains unknown [1,6].

In Tanzania, *S. mansoni* and STH are increasingly becoming major public health concerns, especially among communities living along the Lake Victoria shores, in the North-Western regions of the country [7]. Despite the implementation of a control program in these areas, more than 80% of the school children aged < 15 years are infected with *S. mansoni* and one of the STH species [7-10]. The geographical distribution of these infections has been described in the region by different methods [11,12]. Predictive maps have been generated to guide control programs in the areas but these maps have a limitation in clearly predicting the distribution of these infections due to focal nature of transmission of these infections, especially *S. mansoni* which depends on distribution of its intermediate hosts [11,12]. Thus, there is a paucity of data on the micro-geographical and micro-epidemiological information of these diseases in remote and hard to reach areas [6]. In addition, despite the fact that communities living along the Lake Victoria shores have been known for many years to be highly endemic to *S. mansoni* and STH [7], some have never been reached by control programs, especially the ones residing on the islands of Lake Victoria. Therefore, epidemiological data remain sparse and incomplete. The availability of local epidemiological data would be beneficial for public health authorities and would allow the identification of the high-risk groups and transmission sites. This data would in turn become critical for developing sound and targeted control interventions to reduce the burden of these infections in the rural communities.

In that context, the present study aimed at studying the prevalence of *S. mansoni* and geohelminths and further understanding their associated risk factors in Ukara islands, where there has been up to date inadequate research on the epidemiology of intestinal schistosomiasis and soil-transmitted helminths. Identifying the local risk factors of *S. mansoni* and STH infection represents one step towards a better understanding of the transmission patterns, which will subsequently facilitate the design of cost effective intervention measures.

## Methods

### Study area

Ukara is an island located on the Lake Victoria and is part of the Ukerewe district, Mwanza region, North-Western Tanzania. The island has a total population of 34,181 according to the national census of 2012 [13]. It is divided into four wards, namely Bwisya, Bukungu, Nyamanga and Bukiko. There are eight villages: Bwisya, Nyang’ombe, Bukungu, Chifule, Nyamanga, Chibasi, Bukiko and Kome. There are 12 government-owned primary schools. The main socio-economic activities carried out by the inhabitants of the island include fishing, subsistence farming, livestock keeping and small scale businesses. At the time this study was conducted, no control program was in place against intestinal helminth infections.

### Study design, population and inclusion criteria

A cross-sectional study was conducted among school going children aged 4-15 years focusing on determining the prevalence of infection with STH and *S. mansoni* and their associated risk factors. Children were included in the study if parents/guardians had given written informed consented for them to participate in the study and if assent had been obtained from the children. Teachers were involved to educate parents and children on the importance and risks of participating in the study. Children with a history of taking anthelmintic medication in the past three months were excluded from the study.

### Sample size determination and sampling procedures

The sample size was calculated as described elsewhere [14,15], considering the prevalence of *S. mansoni* infection of 60% in the island of Ukerewe [10], at 95% confidence interval and margin error of 5%. A design effect of 1.5 was considered for the variation in prevalence between schools. A minimum sample of 609 school children was needed for this study.

A simple random sampling method was used to select the villages with a primary school to participate in the study. The number of school children selected from each school was determined by the probability proportional to size of the school and the class population. Systematic sampling, using the class registers as the sampling frame was used, where the names of the children were arranged in alphabetical order. The sampling interval was obtained by dividing the total population in the class with the number of children to be investigated in that class (N/n). After obtaining a start from a table of random numbers, the same interval was kept until the required number of children in each class was obtained.

### Data collection

#### Questionnaire: socio-demographic and assessment of risk factors

A pre-tested Kiswahili translated questionnaire was used to collect demographic, socio-economic activities of parents/guardians, hygiene practices and KAP (knowledge, attitudes and practices). This was done in an attempt to describe the potentially relevant factors associated with the transmission of *S. mansoni* and STH among study participants. The questionnaire was initially developed in English and then translated to Kiswahili and back-translated by a different person who was blinded to the original questionnaire.

#### Stool sample collection and examination of S. mansoni and soil-transmitted helminths

A single stool sample was collected from all study participants. Two Kato Katz thick smears were prepared from different parts of the single stool sample using a template of 41.7 mg (Vestergaard Frandsen, Lausanne, Switzerland) [16], following a standard protocol [16]. Within 30-60 minutes of slides preparation, Kato Katz smears were examined for the presence of STH eggs, specifically hookworms. After 24 hours, the smears were independently examined for *S. mansoni* eggs by two experienced laboratory technicians [16]. For quality assurance, a random sample of 10% of the negative and positive Kato Katz thick smears were re-examined by a third technician.

### Data analysis

The collected data were entered into a data base using Epi data version 3.1. Data analysis was done using STATA version 12.0 (Stata corp, Chicago). The prevalence of *S. mansoni* 95% confidence interval (95% CI) was obtained by binomial logistic regression taking into account clustering by schools. The comparison of prevalence by demographic factors for *S. mansoni* infection was tested for significance using χ^2^ or fisher exact test where appropriate. The age variable was described as mean ± standard deviations. The arithmetic mean of *S. mansoni* egg counts for each participant was calculated from the counts of two Kato Katz thick smears and multiplied by 24 to obtain individuals’ eggs per gram of faeces [17]. *S. mansoni* egg counts were over dispersed so were logarithmically transformed prior to analysis. The geometric mean intensity eggs per gram of faeces (GM-epg) of *S. mansoni* infection were obtained as the antilog of the mean of the transformed egg counts. The comparison of geometric mean egg counts for *S. mansoni* between various demographic factors was undertaken using t-tests and ANOVA. The intensity of infection was categorized as: 1-99 epg, 100-399 epg, ≥400 epg defined as low, moderate and heavy intensities of infection respectively [17]. To determine the factors associated with *S. mansoni* infection and intensities, multiple linear and multivariable logistic regression models were used, controlling for other explanatory variables. The model building strategy was to first identify potential factors at bivariate/linear regressions level and include these factors in the multivariable/multiple linear regression level. Factors with *P*-value < 0.2 were identified at bivariate level and were considered for the final model. Stepwise backward procedures were used to determine whether these variables were independent factors of intensity of *S. mansoni* infection by using adjusted odds ratios (AOR) for linear models and the 95% confidence interval (CI).

### Ethical consideration

Ethical approval was obtained from the joint Ethical and Review Committee of Bugando Medical Centre and Catholic University of Health and Allied Sciences. The study received further clearance from the district administrative authorities (District Education Officer, Medical Officer, Executive Officer) and division authorities at Ukara Island. Kiswahili translated informed assent and consent forms were used to obtain parents/guardians consent for the children to participate in the study and assent of the children. For illiterate parents, a thumb print was used to sign the assent and consent forms after a clear description of the study objectives and their acceptance for their children to participate. All study participants who were infected with *S. mansoni* and STH were treated with praziquantel (40 mg/kg) and albendazole (400 mg) according to WHO and country guidelines [17].

## Results

### Demographic characteristics of the study population

A total of 774 school children aged 4-15 years from five primary schools were enrolled in the study. Of these children, 54.39% (n = 421) were females and 45.61% (n = 353) were males. The mean age of the study participants was 8.85 ± 2.12 years. The majority of the children’s parents reported to be peasants, 72.61% (n = 562), and the remaining were mainly involved in fishing activities. The main source of drinking water for the children was reported to be Lake Victoria, 84.5% (n = 654). Table 1 shows the demographic characteristics of the study participants.

**Table 1:** **Demographic information of school children participated in the study from Ukara Island, North-Western Tanzania (n = 774)**

**Characteristics**	**n**	**%**
**Sex**		
Female	421	54.39
Male	353	45.61
**Age (in years)**		
4 - 7	234	30.23
8 - 10	370	47.80
11 - 15	120	21.96
**Occupational of parents**		
Peasants	562	72.61
Fishing	212	27.39

### Overall prevalence of *Schistosoma mansoni* and soil transmitted helminth infections

Overall, 66.11% (n = 511/773, 95% CI = 62.76 -69.45) of the study participants were infected with at least one of the parasites investigated in the present study. The overall prevalence of *S. mansoni* was 63.91% (n = 494/773, 95% CI = 60.51 - 67.30). The overall prevalence of soil transmitted helminths was 6.73% (n = 52/773, 95% CI = 4.95 – 8.49) with the prevalent species of the STH being hookworm 5.69% (n = 44/773, 95% CI = 4.06 –7.32). Other STH helminth observed was *A. lumbricoides,* 1.03% (n = 8/773, 95% CI = 0.32- 1.75).

### Prevalence of *Schistosoma mansoni* stratified by demographic characteristics

**Figure 1:**
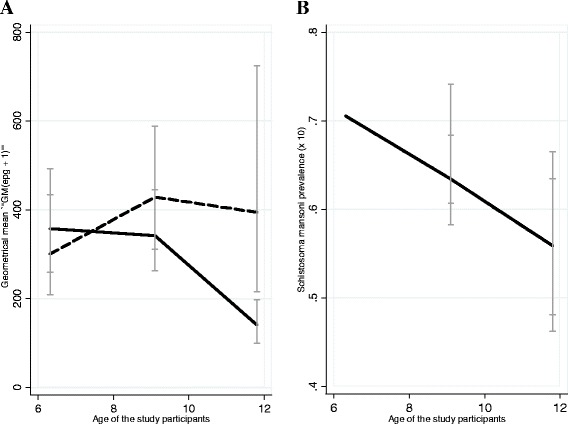
**A:** **Intensities of** ***Schistosoma mansoni*** **infections stratified by age and sex of the study participants (dotted line = Male: Thick/solid line = Female** **B** **: Prevalence of** ***Schistosoma mansoni*** **stratified by age of the study participants.**

The prevalence of *S. mansoni* in relation to the demographic characteristics of the study participants is shown in Table 2. The prevalence of *S. mansoni* did not differ by sex of the study participants (*P =* 0.23) but varied significantly by age groups, with the youngest age group (4 - 10 years) having the highest prevalence (*P <* 0.014) (Figure 1B). The prevalence decreased with increased age of the study participants, with the age group aged 11-15 years having the lowest prevalence. Similarly, the prevalence of *S. mansoni* varied significantly by school location, with schools located along the shorelines of Lake Victoria having the highest prevalence (*P <* 0.0001) (Table 2). The prevalence of the disease also varied significantly by reported parental occupation, with children reporting their parents to be involved in fishing activities having the highest prevalence (*P <* 0.0001) compared to those from farming families. Reported history of regularly visiting (for bathing/swimming/washing clothes or utensils more than three times a week) Lake Victoria was associated with significantly higher prevalence of the disease (*P <* 0.0001) (Table 2).

**Table 2:** **Prevalence of** ***Schistosoma mansoni*** **stratified by demographic characteristics of the study participants**

**Variable**	**No examined**	**Prevalence (%)**	**95% CI**	**χ** ^**2**^	**P–value**
**Sex**					
Female	421	277 (65.80)	46.79 – 92.51	1.43	0.23
Male	352	217 (61.65)	41.90 – 90.69		
**Age (in years)**					
4 – 7	234	165 (70.51)	57.68 – 86.19	9.2117	<0.010
8 – 10	370	234 (63.41)	40.94 – 98.22		
11 – 15	120	95 (55.88)	39.91 – 78.22		
**Schools**					
Chifule	190	145 (76.32)	69.70 – 83.56		
Mubule	155	98 (63.23)	56.97 – 70.17		
Kome	149	144 (96.64)	93.72 – 99.57	190.18	<0.001
Nyamanga	155	81 (52.26)	44.70 – 61.09		
Kumambe	124	26 (20.97)	12.59 – 34.91		
**Parents occupation**					
Peasants	561	327 (58.29)	52.65 – 64.53	27.9905	<0.001
Fishing	212	167 (78.77)	76.96 – 80.63		
**Presence of toilet at home/school**					
Yes	675	427 (63.26)	56.91 – 70.32	0.97	0.33
No	98	67 (68.37)	65.15 – 71.73		
**Lake visit**					
Always	653	442 (67.69)	64.04 – 70.62	26.07	<0.001
Sometimes	120	52 (43.33)	26.59 – 70.62		
**Paddy cultivation**					
Always	128	80 (62.5)	53.99 – 71.00	0.13	0.717
Sometimes	645	414 (64.19)	60.47 – 67.89

### Intensity of *Schistosoma mansoni* and soil-transmitted helminths

The overall geometrical mean egg per gram of faeces (GM-epg) for individuals with detectable *S. mansoni* eggs was 323.41epg (95% CI: 281.09 – 372.11). Males had higher infection intensities (371.97epg, 95% CI: 297.8-465.7) than females (289.84epg, 95% CI: 242.19-346.88, *P <* 0.0013) (Table 3) (Figure 1A). The intensity of infection varied significantly across the schools which children attended (*P <* 0.0001), with the schools located along the shorelines of Lake Victoria having the highest intensities (Table 3). A history of regularly visiting (for bathing/swimming/washing clothes or utensils more than three times a week) Lake Victoria was also associated with the highest intensity of infection (*P <* 0.04).

**Table 3:** **Intensity of** ***S. mansoni*** **infection stratified by demographic factors of the study participants**

**Variables**	**Number**	**GM-EPG**	**95% CI**	***P*** **-value**
**Overall**	494	323.41	281.09-372.11	
**Sex**				
Male	217	371.97	297.77- 464.65	0.013*
Female	277	289.84	242.19-346.88	
**Age (in years)**				
4 – 7	165	329.64	259.29 – 419.08	0.52**
8 – 10	234	376.64	307.69 – 461.03	
11 - 15	95	214.98	154.65 – 298.84	
**Schools**				
Chifule	145	481.71	368.59-629.54	<0.001**
Mubule	98	153.39	126.17-186.51	
Kome	144	701.35	545.33-902.00	
Nyamanga	81	136.98	99.24-189.07	
Kumambe	26	116.49	88.17-153.89	
**Parents occupation**				
Peasants	327	282.90	238.32-335.36	0.099
Fishing	167	420.30	328.98-536.98	
**Presence of toilet at home/school**				
Yes	427	320.37	276.04-371.82	0.38*
No	67	343.50	225.51-523.23	
**Lake visit**				
Always	442	350.94	302.40-407.26	0.04*
Sometimes	52	161.53	110.45-236.24	
**Paddy cultivation**				
Always	80	267.04	197.73-360.66	0.12**
Sometimes	398	344.38	293.39-404.22	
Not real	16	176.69	76.34-408.98	

*P*-values = t-test* and Anova**.

GM-epg = Geometrical Mean egg per gram of feaces.

Of all the children found infected with any of the STH observed in the study, the majority had a light to moderate intensity of infection.

### Factors associated with *Schistosoma mansoni* infection and intensity

The results of bivariate and multivariable analysis for the factors associated with *S. mansoni* infection are shown in Table 4. At bivariate level, a young age group (*P <* 0.014), parental fishing occupation (*P <* 0.0001), location of the schools along the shorelines of Lake Victoria (*P <* 0.0001) and reported history of visiting Lake Victoria (*P <* 0.0001) remained significantly associated with *S. mansoni* infection. However, on multivariable analysis, only the location of schools which children attended remained associated with *S. mansoni* infection (*P <* 0.0001).

**Table 4:** **Factors associated with** ***Schistosoma mansoni*** **infection among school children in Ukara Island, North-Western Tanzania**

**Variable**	*COR	**95% CI**	***P*** **-value**	**AOR	**95% CI**	***P*** **-value**
**Sex**						
Female	1	-	-	-	-	-
Male	0.83	0.62-1.12	0.23	0.89	0.63-1.26	0.51
**Age (in years)**						
4 - 7	1.37	0.95 – 1.98	0.096	1.15	0.74 – 1.79	0.53
8 – 10	1.89	1.25 – 2.85	0.003	1.28	0.79 – 2.09	0.35
11 - 15	1			1		
**Parents occupation**						
Peasants	1	-	-	-	-	-
Fishing	2.66	1.84-3.84	<0.001	1.49	0.98 - 2.59	0.061
**Schools**						
Kumambe	1	-	-	-	-	-
Nyamanga	4.13	2.42-7.05	<0.001	3.89	2.24 – 6.74	<0.001
Kome	108.55	40.29-292.41		93.26	33.82 - 257.19	
Mubule	6.48	3.77-11.14		6.15	3.46 – 10.95	
Chifule	12.15	7.03-20.98		10.15	5.59 – 18.38	
**Lake visit**						
No	1	-	-	-	-	-
Yes	2.74	1.84-4.07	< 0.001	1.03	0.66-1.71	0.81
**Paddy cultivation**						
No	1	-	-	-	-	-
Yes	1.69	0.83-3.49	0.15	1.92	0.85-4.29	0.12

*COR = Crude Odd Ratio **AOR = Adjusted Odd Ratio CI = 95% confidence Interval.

The results of multiple linear regression revealed that fishing as parents occupations (AOR = 1.20, 95% CI; 1.02-1.42, *P <* 0.03), reportedly involved in paddy cultivation (AOR = 1.45,95% CI: 1.004-2.10, *P <* 0.048) and the location of the schools at Nyamanga (AOR = 1.75,95% CI; 1.37 – 2.24, P < 0.0001), Kome (AOR = 7.52, 95% CI; 5.78-9.75, *P <* 0.0001), Mubule (AOR = 2.22,95% CI; 1.73 – 2.86, *P <* 0.0001) and Chifule (AOR = 3.91,95% CI; 3.03-5.04, P < 0.0001) villages *P <* 0.0001) along the shorelines of Lake Victoria remained significantly associated with the intensity of *S. mansoni* infection (*P <* 0.0001) Additional file 1. The prevalence of STH was very low to allow the analysis of factors associated with STH in the present study population.

## Discussion

### Prevalence of *Schistosoma mansoni* and soil-transmitted helminth infection

The overall prevalence of *S.mansoni* observed in the present study was similar to the prevalence of 64.3% reported among school children along the Lake shore in Sengerema district, North-Western Tanzania [8], but slightly higher compared to studies in Mbita Island in Western Kenya (60.5%) [18] and Sesse islands on Lake Victoria in Uganda (58.1%) [19]. In addition, the prevalence and intensity of *S. mansoni* infections varied significantly by demographic characteristics of the study participants. The prevalence and intensity of infection varied by age of the study participants, gender, village of residence and parental occupation. In endemic areas, it is usually acknowledged that *S. mansoni* intensities of infection show a peak at the age group 6-19 years and, thereafter, the intensities decline gradually with an increased age [20-22]. Similarly, in the present study, the youngest age groups had the highest intensity of infection. Our study further showed that in the study area, infection with *S. mansoni* starts at a young age (probably < 4 years of age). If these children are not treated in time [22], by the time they start school, they may have developed significant morbidities [10].

We observed an inverse relationship between the proximity to the lake and *S. mansoni* prevalence and intensity of infection in the study areas, with schools located closest to Lake Victoria having the highest prevalence compared to schools which were located away from the Lake shores. Our observations were consistent with the results of Handzel *et al* who observed the decrease in prevalence of *S. mansoni* with increasing distance from the Lake Victoria shore [23]. The school located nearest the lake (750 meters) had a mean prevalence of 80% which decreased to 20% at a distance of 4-13 km from the lake shore [23]. Similarly, in Mbita and its adjacent islands, schools located in close proximity to Lake Victoria had the highest prevalence of *S. mansoni* [18,24,25].

The overall prevalence of soil-transmitted helminths observed in the present study population was very low compared to 12.4% [18], 16.2% [26] and 42.5%% [23] reported from Western Kenya. Of all the soil-transmitted helminths observed in the present study, hookworms were the most predominant species, although their prevalence was very low as compared to data reported by previous studies in North-Western Tanzania which quoted a prevalence of 38% [8] and 37% [27] and in Western Kenya (42.5%) [23]. Along the Lake Victoria shores in Western Kenya and North-Western Tanzania, hookworms appear to be the predominant species and other soil-transmitted helminths areas are rarely found. Two previous studies in North-Western Tanzania reported a prevalence of <1% of *A. lumbricoides, T. trichiura* and *E. vermicularis* [8,27]. The high tolerance of hookworms’ eggs and larval stages to the variation of the soil temperature has been described as a key factor for the high transmission and prevalence of these parasites in the area [12].

The majority of the infected children had a light to moderate infection intensity of these helminths infections. This observation was consistent with data from previous studies which observed that most of the individuals infected with soil-transmitted helminths normally excrete a low number of eggs [18,24-26].

### Intensity of *S. mansoni* infections

Epidemiological surveys along the Lake Victoria have shown that school children are carrying the highest intensity of infection as compared to other age groups [9,18] and male individuals are more heavily infected [9,28]. In the present study, the majority of the study participants infected with *S. mansoni* had light to moderate intensities and only a few were heavily infected with *S. mansoni*. Male individuals had the highest infection intensity as compared to female individuals. The difference in intensity of infection between sexes is mainly associated with the variation of exposure to risk areas and the time spent in water sources [29]. Male individuals tend to spend more time in water sources compared to female individuals [30]. A high exposure is associated with swimming and sometime fishing in male children and can result in the maintenance of a high prevalence and intensity of infection into adulthood [9,30].

The focal nature of *S. mansoni* transmission along the present study area appears to influence intensity of infection. We have observed variations in the intensity of infection between schools, with schools located along the lake shores having the highest intensity of infection as compared to schools which were located away from the lake. Similar findings have been reported in Ssese island on the Lake Victoria shore in Uganda [19] and in Western Kenya [18,23-25].

### Risk factors associated with intensity and *S. mansoni* infection

In *S. mansoni* endemic areas, gender, age group, geographical location and occupation are some of the well-described demographic factors reported to be associated with infection and intensities [29,31-33]. Similarly, our findings showed that *S. mansoni* infection was mainly associated with the younger age group (4 - 10 years). In addition, parental occupation, especially involvement in fishing, the location of the school and a reported history of visiting the lake regularly were significantly associated with *S. mansoni* infection. However, on a multivariable analysis, only the location of schools remained associated with *S. mansoni* infection. As explained above, the proximity to the lake shores was associated with an increased risk of high infection in the present study area. In Sesse Island, in Uganda, a combination of malacological and parasitological surveys revealed that *S. mansoni* infection was only occurring in certain parts of the island [19]. The same applied in Western Kenya [18,34]. The present study did not include malacological surveys and these surveys are recommended in future studies in the area.

Conversely, a multiple linear regression model revealed that being male and the location of the schools along the shorelines of Lake Victoria remained significantly associated with the intensity of *S. mansoni* infection. Similar results have been described elsewhere in sub-Saharan Africa [10,35]. A heavy intensity of infection was mainly seen in male individuals. The schools location along the shorelines of Lake Victoria mainly defines the level of exposure and transmission of the disease in the study area. Children attending schools located in villages which had close proximity to the lake, had highest intensities and appeared to be more exposed to cercariae infested water as compared to those who were living away from the lake shore [18,24,25].

## Conclusion

*Schistosoma mansoni* infection is highly prevalent in the Ukara Island whereas the prevalence of soil-transmitted helminths is low. The risk of infection with *S. mansoni* and the intensity increased along the shorelines of Lake Victoria. These findings reveal an actual presence of intestinal schistosomiasis in remote areas which have not been covered by any control program. Moreover, these findings call for the need to urgently implement integrated control interventions covering school going children of all ages, starting with targeted mass drug administration in relation to specific location of the villages.

## Additional file

Additional file 1: Table S4. Results from multivariate analysis controlling for random effects of villages/schools.
