# Supplementary material for: Intestinal schistosomiasis and geohelminths of Ukara Island, North-Western Tanzania: prevalence, intensity of infection and associated risk factors among school children
Source: Parasit Vectors. 2014 Dec 23;7:612. doi: 10.1186/s13071-014-0612-5 (PMC4297386; doi:10.1186/s13071-014-0612-5)
Supplement: Additional file 1: Table S4. — Results from multivariate analysis controlling for random effects of villages/schools. [file 13071_2014_612_MOESM1_ESM.doc]

**Additional file 1: Table S4**: Results from multivariate analysis controlling for random effects of villages/schools

| **Variables** | **AOR** | **95%CI** | ***P*-value** |
| --- | --- | --- | --- |
| **Sex** | | | |
| Female | 1 |  | 0.51 |
| Male | 1.13 | 0.57 – 1.26 |
| **Age (in years)** | | | |
| 4 – 7 | 1.27 | 0.29 – 2.07 | 0.35 |
| 8 – 10 | 1.15 | 0.35 – 1.75 | 0.23 |
| 11 - 15 | 1 |  |  |
| **Location of schools** | | | |
| Kumambe | 1 |  | <0.0001 |
| Nyamanga | 3.89 | 2.24 – 6.74 |
| Kome | 93.69 | 78.44 – 257.24 |
| Mubule | 6.17 | 3.46 – 10.91 |
| Chifule | 10.18 | 5.58 – 18.35 |
| **Parent occupations** | | | |
| Peasants | 1 |  | 0.06 |
| Fishing | 1.48 | 0.02 – 2.25 |
| **Lake visit** | | | |
| No | 1 |  | 0.81 |
| Yes | 1.06 | 0.51 – 1.70 |
| **Involved in paddy cultivation** | | | |
| No | 1 |  | 0.12 |
| Yes | 1.92 | 0.18 – 4.29 |

**Key:** AOR= Adjusted Odd Ratio, CI=Confidence Interval,
